# The Shaping of AMPA Receptor Surface Distribution by Neuronal Activity

**DOI:** 10.3389/fnsyn.2022.833782

**Published:** 2022-03-21

**Authors:** Thomas Edward Chater, Yukiko Goda

**Affiliations:** Laboratory for Synaptic Plasticity and Connectivity, RIKEN Center for Brain Science, Wako-shi, Japan

**Keywords:** AMPAR, synaptic plasticity, glutamate receptor, glutamatergic, synapse

## Abstract

Neurotransmission is critically dependent on the number, position, and composition of receptor proteins on the postsynaptic neuron. Of these, α-amino-3-hydroxy-5-methyl-4-isoxazole propionic acid receptors (AMPARs) are responsible for the majority of postsynaptic depolarization at excitatory mammalian synapses following glutamate release. AMPARs are continually trafficked to and from the cell surface, and once at the surface, AMPARs laterally diffuse in and out of synaptic domains. Moreover, the subcellular distribution of AMPARs is shaped by patterns of activity, as classically demonstrated by the synaptic insertion or removal of AMPARs following the induction of long-term potentiation (LTP) and long-term depression (LTD), respectively. Crucially, there are many subtleties in the regulation of AMPARs, and exactly how local and global synaptic activity drives the trafficking and retention of synaptic AMPARs of different subtypes continues to attract attention. Here we will review how activity can have differential effects on AMPAR distribution and trafficking along with its subunit composition and phosphorylation state, and we highlight some of the controversies and remaining questions. As the AMPAR field is extensive, to say the least, this review will focus primarily on cellular and molecular studies in the hippocampus. We apologise to authors whose work could not be cited directly owing to space limitations.

## Introduction

### AMPAR’s Place in the Synaptic Receptor Complement

The majority of excitatory synapses in the mammalian brain are glutamatergic. Presynaptic activity drives the fusion of vesicles packed with glutamate, which then diffuses across the ~20 nm synaptic cleft and binds to a set of proteins on the postsynaptic surface belonging to the glutamate receptor family. These receptors consist of the ionotropic family members including AMPARs, N-methyl-D-aspartate receptors (NMDARs), and kainite receptors (KARs), that have been classified according to their agonist selectivity (Hollmann and Heinemann, 1994; Dingledine et al., 1999), and the metabotropic glutamate receptor (mGluR) family. The ionotropic glutamate receptor family members are broadly specialized, with AMPARs as the main mediator of excitatory synaptic transmission, NMDARs being required for plasticity induction, and KARs for modulation. Multiple methods have been employed to directly count the number of receptors at a particular synapse (Patrizio and Specht, 2016), see Box 1 for methods to measure AMPARs in neurons. Whilst the number varies depending on brain region and synapse type, an average mammalian cortical synapse may contain on the order of ~20–30 AMPARs, accompanied by 1–10 NMDARs (Racca et al., 2000; Masugi-Tokita et al., 2007; Nair et al., 2013). Spatially, NMDARs form clusters at the centre of the postsynaptic density (PSD), surrounded by clusters of AMPARs, with a diffuse arrangement of mGluRs (Goncalves et al., 2020; Li et al., 2021).

Box 1 **Methods of studying AMPARs.**

*Antibody Labelling—Fixed Samples*

One of the classic methods to visualize AMPARs. Many studies have labelled endogenous receptors both in cultures and brain slices. Both subtype-specific and phospho-specific labels are widely used. Compatible with electron microscopy for precise localisation. Temporal precision is limited to the time of fixation.

*Antibody—Live-Labelling and Imaging*

Antibodies can be conjugated to various tags such as Alexa dyes, quantum dots, and others, allowing direct imaging of surface AMPAR populations in live, behaving cells. There has been some speculation as to whether larger tags can successfully access the synaptic cleft (Lee et al., 2017). This technique continues to advance, with brighter and smaller tags becoming available.

*Protein/Peptide Tagged Receptors*

One method of optically visualising AMPARs is to fuse a fluorescent protein to the subunit (GFP, YFP, etc.) followed by exogenous expression or replacing the endogenous protein. This technique has been further enhanced by the use of super-ecliptic pHluorin. This pH-sensitive GFP variant is quenched at the lower pH found in endosomes, and fluoresces brightly when exocytosed onto the cell surface (Miesenböck et al., 1998). Similar to quantum dots/antibody labels, there is some worry that the tag may restrict the receptor movement. Knock-in mice for pHluorin-tagged GluA1 and GluA2 have been developed. Protein/peptide tagging of endogenous receptors is also possible with CRISPR/Cas9 techniques like vSLENDR (Mikuni et al., 2016; Nishiyama et al., 2017).

*Microscopy*

Using the tagged receptor approach (quantum dot/dye/XFP etc.), many groups have capitalised on highly sophisticated microscopes to image AMPARs at synaptic or subsynaptic resolution. Confocal (Ashby et al., 2004), 2-photon (Makino and Malinow, 2011), TIRF (Tanaka and Hirano, 2012), STORM (Xu et al., 2020), STED (Nair et al., 2013), and uPAINT (Giannone et al., 2010) have all been used to image populations of AMPARs at living synapses.

*Electrophysiology*

Many studies use recordings of either evoked or spontaneous synaptic activity in order to assess synaptic AMPAR content. A common technique is to record miniature excitatory postsynaptic currents (mEPSCs) with the amplitude being a readout of the abundance of functional postsynaptic AMPARs (i.e., postsynaptic strength). Under physiological recording conditions mEPSC amplitude consists mainly of AMPAR-mediated current, but with some NMDAR contribution. Notably, whether mEPSCs reflect the same population of AMPARs that respond to action potential-triggered release remains a point of contention. Although mEPSC provides no spatial information regarding its source, for typical recordings, mEPSC waveform gives some clue to the dendritic origin with respect to the relative distance from the soma.

Upon synaptic activation and the release of glutamate from the presynaptic neuron, AMPARs open, thereby allowing Na^+^ to flow down its electrochemical gradient into the postsynaptic neuron. This depolarizes the postsynaptic membrane and relieves the Mg^2+^ block of nearby NMDARs, which then allow Ca^2+^ to enter the neuron if activity levels have been high enough (Traynelis et al., 2010). But within this simple description lies the vastly complex molecular machine of the postsynapse. Whilst postsynaptic density (PSD) and NMDAR complexes play key parts, this article will feature AMPARs as the essential elements of the postsynapse.

The 4 AMPAR subunits (GluA1–4) are encoded by the genes *Gria1–4*, and each plays a slightly different role in the mammalian central nervous system (Hollmann and Heinemann, 1994). GluA4 is expressed early in development (Monyer et al., 1991). As circuits mature, spontaneous activity drives GluA4-containing AMPARs into synapses, and they are gradually replaced by GluA2-containing subunits (Zhu et al., 2000). Some GluA4 survives into adulthood, as measured in both murine and human brain tissue (Allen Institute for Brain Science, 2004; Kawahara et al., 2004; Lein et al., 2007; Oh et al., 2014; Daigle et al., 2018; Harris et al., 2019). Synaptic and surface AMPARs in the adult hippocampus are largely composed of GluA1–3 that form predominately GluA1/2 and GluA2/3 heterodimers, with GluA1/2 being the most common (Wenthold et al., 1996; Zhu et al., 2000; Mansour et al., 2001; Kessels and Malinow, 2009). Neurons in which the genes encoding GluA1–3 (i.e., *Gria1–3*) have been deleted have no synaptic AMPARs at all, suggesting that GluA4 cannot compensate after its developmental downregulation (Lu et al., 2009).

It has been known for decades that neuronal activity can drive fast changes in synaptic strength that are long-lasting in nature (Bliss and Lomo, 1973; Dudek and Bear, 1992). These have been broadly divided into stimuli that potentiate or depress populations of synapses. Brief bursts of tetanic activity (i.e., ≈100 Hz for seconds) cause synapses to increase in strength over the period of seconds or minutes, and these increases last for up to 10 h in acute slice preparations (Redondo et al., 2010). On the flip side, longer trains of low-frequency activity (i.e., 1 Hz for minutes) typically elicits a fast-acting weakening of synaptic strength. These two opposing forms of synaptic plasticity, LTP and LTD, have been extensively studied over decades, and although some controversies still exist, trafficking of AMPARs at the synapse is the main postsynaptic substrate for these changes. However, patterns of neuronal activity are diverse, and the neuronal and synaptic responses to changes in activity levels are complex and interesting. Let us begin with basal behaviour.

### Constitutive Trafficking of AMPARs and Baseline Surface Mobility

Synapses are not static structures. Befittingly, AMPARs are constantly on the move, even in the absence of neuronal activity (Ehlers et al., 2007). AMPARs constitutively traffic from endosomes to the cell surface with a half-life of 1–2 days (Archibald et al., 1998; O’Brien et al., 1998; Ojima et al., 2021). Surface delivery of AMPARs occurs in a subunit specific manner (Passafaro et al., 2001; Shi et al., 2001) and is modulated by accessory proteins such as GRIPs and the transmembrane AMPAR accessory proteins (TARPs; reviewed in Bissen et al., 2019) and members of rab GTPases (see Hausser and Schlett, 2019 for review). Once on the surface, AMPARs laterally diffuse in the plane of the membrane and enter synapses, where interactions with a host of proteins facilitate their retention (Dong et al., 1997; Gardner et al., 2005; Bats et al., 2007; Schwenk et al., 2009; see Kamalova and Nakagawa, 2021 for review) and help align AMPARs with presynaptic release sites (Biederer et al., 2017).

**Figure 1:**
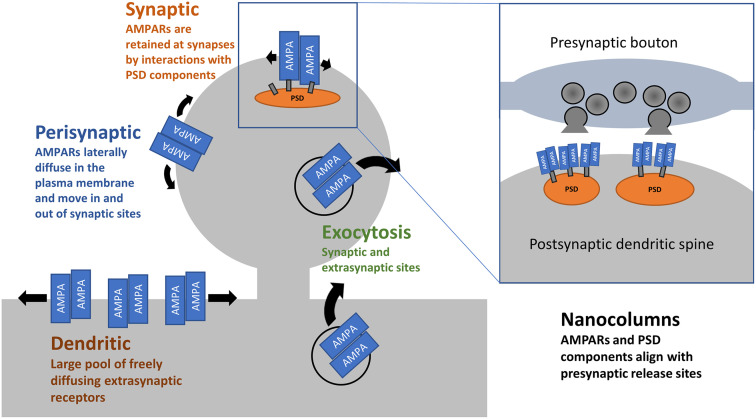
AMPAR trafficking and distribution. AMPARs arrive at the cell surface through exocytosis at dendritic, presynaptic, and synaptic sites. This is both constitutive and triggered by activity. Once on the cell surface, AMPARs are free to laterally diffuse in the plane of the plasma membrane. At synaptic sites, interactions between AMPARs, their accessory proteins, and PSD components act to retain receptors. Within synapses, there is precise alignment between postsynaptic AMPARs and the presynaptic release sites.

Even on millisecond timescales, the movement of AMPARs can affect synaptic transmission. Upon binding glutamate, AMPARs undergo desensitization within tens of milliseconds, and this contributes to a short-term depression of synaptic strength (Twomey et al., 2017). A series of studies has demonstrated that lateral movement of AMPARs in and out of synapses, on the order of milliseconds, can partially offset the drop in transmission caused by desensitisation by supplying non-desensitized receptors (Heine et al., 2008). Freezing AMPARs in place with cross-linking antibodies or overexpression of AMPAR-binding PSD proteins prevented the partial rescue, as did over-expression of AMPAR subunits incapable of binding their PSD partners. Preventing this fast lateral diffusion of AMPARs *in vivo* leads to deficits in LTP and hippocampal-dependent contextual fear memory (Penn et al., 2017). Notably, deficits in AMPAR surface diffusion have been implicated in animal models of depression/stress involving corticosterone (Groc et al., 2008) and in neurological disorders such as Huntington’s disease (Zhang et al., 2018). Collectively, these reports highlight the importance of the pool of extrasynaptic AMPARs across the dendritic surface (Opazo et al., 2012; Nair et al., 2013; Lee et al., 2017, see Figure 1) in maintaining efficacious synaptic transmission with behavioural consequences.

The cell surface distribution of AMPARs heavily depends on their subunit composition. GluA1 consistently displays a punctate and synaptic distribution when compared to GluA2, which is much more diffuse, with a greater proportion of the population being extrasynaptic (Tian et al., 2015). Importantly, the estimates of the relative abundance of synaptic and extrasynaptic AMPARs can vary depending on the specifics of the method employed as shown by Lee et al. (2017). In this study, the size of the quantum dot (QD) attached to the AMPAR to monitor its mobility strongly affected the ability of the receptor to be trapped in synaptic sites, with large QDs massively decreasing the population of synaptic AMPAR and driving a corresponding increase in the extrasynaptic pool. This study suggests that in fact ≈84%–97% of AMPARs are usually resident within synaptic sites, in contrast to previous studies. The age of preparation is critical too; the extrasynaptic pool of receptors becomes stably maintained as synaptogenesis ramps up (Cottrell et al., 2000).

The pattern of AMPAR surface diffusion is modulated by synaptic activity. For example, the synaptic resident time for GluA1 is dependent on activity levels. Synapses formed with a presynaptically silenced input that expressed tetanus toxin to block the release of neurotransmitters via the cleavage of VAMP2/synaptobrevin contained reduced steady-state levels of GluA1 with no effect on GluA2 levels (Harms and Craig, 2005; Harms et al., 2005); upon examining receptor dynamics, such inactive synapses did not retain GluA1 (Ehlers et al., 2007). In contrast, at control active synapses, GluA1 was diffusionally confined, whereas, in extrasynaptic regions on the dendritic surface, GluA1 showed elevated surface diffusion rates (Ehlers et al., 2007). Globally silencing the cultures by preventing spiking [with tetrodotoxin (TTX), a sodium channel blocker] and blocking synaptic glutamate receptors with competitive antagonists [2-amino-5-phosphonovaleric acid (APV) for NMDARs and 6-cyano-7-nitroquinoxaline-2,3-dione (CNQX) for AMPARs] for 4 h had no additional effect on GluA1 diffusional patterns, thereby suggesting the contribution of a competitive process between active and inactive synapses for GluA1 recruitment. A recent study (Hussain and Davanger, 2015) replicated the impaired synaptic retention of GluA1 upon silencing of presynaptic neurotransmitter release with tetanus toxin but attributed the effect to the block of surface delivery of endosomes containing GluA1 and (postsynaptically located)VAMP2. GluA2 on the other hand showed a small but significant increase in surface levels. Moreover, acutely blocking neurotransmitter vesicle filling with bafilomycin did not alter GluA1 levels but reduced GluA2 levels. Béïque et al. (2011) used two photon glutamate uncaging to directly measure AMPA currents at dendritic spines as opposed to an axon whose activity was suppressed by the exogenous expression of Kir2.1. Kir2.1 is an inwardly-rectifying potassium channel that has been used to hyperpolarize neurons and impair their spiking activity. Spines opposite these silenced inputs showed an increase in the total AMPAR component. Moreover, in contrast to studies that used tetanus toxin, the authors reported a specific increase in GluA2-lacking AMPARs that suggested the increase in synaptic GluA1, and that this upregulation required activity-regulated cytoskeleton-associated protein Arc (also called Arg3.1), a member of the immediate-early gene family. This increase was specific to the spine, as neighbouring spines on the same dendrite were not affected. Interestingly, in these experiments, the correlation between spine size and AMPAR complement was disrupted. Altogether, a better understanding of how the timing and the mode of presynaptic activity influence the property of postsynaptic AMPAR subtypes is warranted.

The functions of the extrasynaptic pool, which is likely multi-faceted, also remain to be clarified. Does it simply provide a reserve for quickly replenishing desensitised receptors at active synapses or for recruiting new receptors at synapses undergoing potentiation? Does it play a role in the formation of new postsynaptic structures? To what extent do perisynaptic receptor complexes detect glutamate spillover from active synapses? Interestingly, in a model of hepatic encephalopathy, neurons preserve their synaptic AMPAR population at the expense of the extrasynaptic pool (Schroeter et al., 2015). Even when half of the surface AMPARs were lost, synaptic currents as measured by mEPSCs were unaffected, demonstrating that at least one role of extrasynaptic AMPARs may be to act as a buffer against pathological loss of AMPARs.

While the extrasynaptic AMPARs serve as a source of synaptic AMPARs, trafficking of the extrasynaptic pool is likely regulated differently than AMPARs that are delivered directly *via* synaptic or perisynaptic insertion. Particularly, LTP-inducing stimuli cause the fast exocytosis of AMPARs, increasing postsynaptic responses (Malenka and Bear, 2004). The exact site of receptor exocytosis following LTP induction has been long debated with evidence for perisynaptic delivery of AMPARs (e.g., Yang et al., 2008), dendritic exocytosis (e.g., Yudowski et al., 2007; Patterson et al., 2010), and direct synaptic trafficking of receptors (e.g., Patterson et al., 2010). Given that both AMPAR trafficking and surface diffusion are influenced by neuronal activity, it is expected that shifts in the relative balance of the contributing mechanisms underlie the plastic changes in the abundance of synaptic AMPARs. One way to monitor AMPAR trafficking is to tag individual subunits with a pH-sensitive form of GFP [super-ecliptic pHlurorin (“sep”) Miesenböck et al., 1998]. This molecule is quenched in acidic environments, such as those found within intracellular vesicles, but bright when exposed to extracellular milieu with near-neutral pH. A recent study using sep fused to GluA1 (sep-GluA1) to monitor its dynamics found that overexpression of an AMPAR accessory protein [transmembrane AMPAR regulatory protein (TARP)γ-8] reduced constitutive GluA1 endocytosis at extrasynaptic sites but not at synapses, with the overall effect of promoting the surface lifetime of extrasynaptic GluA1 (Harb et al., 2021). Interestingly, removing TARPγ-8 from neurons blocks LTP (Rouach et al., 2005), again potentially linking the requirement for an extrasynaptic pool of receptors for the expression of plasticity. Furthermore, a reserve pool of AMPARs is crucial for LTP regardless of the AMPAR subtype (Granger et al., 2013; discussed below).

As mentioned above, GluA1-containing AMPARs show a tighter synaptic localization compared to GluA2-containing AMPARs that appear to be the main constituents of the extrasynaptic AMPAR pool. Hippocampal neurons also contain a significant amount of GluA3 (Kessels et al., 2009; Renner et al., 2017). However, the role of GluA3 is much less well understood. *Gria3* deletion appears not to affect LTP or LTD (Meng et al., 2003) and memory (Humeau et al., 2007), and under basal conditions, GluA3-containing receptors contribute little to synaptic activity (Meng et al., 2003; Lu et al., 2009). Moreover, a series of seemingly contradictory results has mired the interpretation of the role of GluA3. *Gria3* mRNA levels in the hippocampus are much lower than mRNAs for both GluA1 and GluA2 (Tsuzuki et al., 2001). However, at the protein level GluA3 is abundant (Schwenk et al., 2014), and GluA1/2 and GluA2/3 heterodimers appear to be expressed on the surface in a similar proportion (Kessels et al., 2009), even though GluA2/3 AMPARs contribute little to synaptic and extrasynaptic currents (Lu et al., 2009). What, then, is the purpose of GluA3-containing AMPARs? A recent study has demonstrated that the channel activity of GluA3-containing AMPARs can be switched from a low open probability state to a higher open probability state. This is triggered by the activation of β-adrenergic receptors, leading to increased PKA/Ras activity and elevated cAMP, thereby increasing synaptic strength; however, the exact mechanism remains to be elucidated (Renner et al., 2017). GluA3 has also been demonstrated to contribute to ultrafast kinetics of AMPARs at subclasses of synapses (Antunes et al., 2020). Therefore, GluA3 may serve a specialized function whose activity is tuned to the particular needs of the circuit involved.

### Subsynaptic Structure and Its Dynamics

The recent rise of super-resolution imaging in live neurons has helped reveal the precise location of synaptic AMPAR complexes within the PSD. Multiple studies have demonstrated the existence of synaptic nanodomains or nanocolumns (NCs; see Figure 1), in which postsynaptic AMPARs and other PSD constituents are aligned with the presynaptic active zone components (MacGillavry et al., 2013; Nair et al., 2013; Tang et al., 2016). Such nanoscopic cluster assemblies include the postsynaptic scaffolding proteins PSD-95, Shank3, Homer1c, and GKAP, with GluA2 being enriched in PSD-95 subsynaptic domains (MacGillavry et al., 2013). Across the synapse, presynaptic RIM1/2 forms NCs in alignment with GluA2 and PSD-95 (Tang et al., 2016). NCs, at least those observed postsynaptically, undergo constant remodelling on minute-to-minute timescales and are responsive to various forms of plasticity (MacGillavry et al., 2013). Both chemical LTP or LTD induction causes reorganisation of NCs, with LTP leading to an enrichment of PSD-95 within NCs, and LTD disrupting PSD-95 NCs (Tang et al., 2016; see Biederer et al., 2017, for review). NCs are also sensitive to global changes in network activity studied in cultures. Treatments that trigger homeostatic scaling up or down of synaptic strength (see below) produced a bidirectional change in PSD-95 cluster areas, with TTX-mediated network silencing causing an increase in a cluster area, and activity increase following bicuculline treatment causing a decrease in PSD-95 cluster area (MacGillavry et al., 2013).

How are NC structures maintained? The diffusional confinement of AMPARs that contribute to the NC organization is likely mediated by interactions with PSD scaffold proteins, although other possibilities have also been explored. For example, the “picket-fence” model postulates a set of proteins that form a barrier around the synapse, restricting diffusion in and out of these sites. Concentrations of actin close to the membrane may prevent free diffusion through the fence. It may be that both direct and indirect interaction mechanisms contribute to AMPAR synaptic trapping (Nair et al., 2013). Altering the levels of PSD-95 by overexpression or knockdown alters the properties of synaptic AMPAR nanodomains, suggesting a key role for this PSD component in organizing and maintaining NCs. The dynamics of PSD-95 provides further insights into the postsynaptic organization and function. Upon triggering LTP, PSD-95 is phosphorylated and transiently exits dendritic spines, allowing for LTP expression (Steiner et al., 2008). Overexpression of PSD-95 occludes LTP (Ehrlich and Malinow, 2004), and mice lacking PSD-95 have enhanced LTP, no LTD, and show memory deficits (Migaud et al., 1998). PSD-95 turns over rapidly, diffuses in and out of spines and exchanges with neighbouring spines (Gray et al., 2006). The synaptic retention time of PSD-95 is modulated by activity, increasing during development, and dropping significantly following sensory deprivation (Gray et al., 2006).

Other PSD components including PSD-93 and SAP102, also contribute to AMPAR synaptic localization *via* AMPAR C-terminal tail interactions (Elias et al., 2006, 2008). AMPAR N-terminal domain interactions could also contribute to NCs. GluA1 and GluA2 extracellular interactions have been shown to help anchor AMPARs at synaptic sites (Watson et al., 2017) and regulate presynaptic structure (Ripley et al., 2011). For example, GluA2 interacts with N-cadherin through its N-terminal domain to regulate dendritic spine formation and presynaptic release (Saglietti et al., 2007; Vitureira et al., 2011). As mentioned above, AMPAR synaptic localization is also promoted by binding to other accessory proteins such as TARPs (Chen et al., 2000; Schnell et al., 2002; Bats et al., 2007), and thus NCs are subject to complex regulation.

### Activity-Triggered Fast Exo- and Endocytosis of AMPARs

We have thus far discussed activity-dependent changes in synaptic AMPARs mainly in the context of the relative contributions of extrasynaptic and synaptic AMPARs. Here we present progress on the topic of regulation of synaptic AMPAR complement by focusing on exo-endocytic traffic. While AMPARs are fairly free to diffuse along the cell surface, various studies have predicted, or directly shown, that the narrow spine neck acts as a diffusional barrier for spine entry (Ashby et al., 2006; Jaskolski et al., 2009). This works in both directions, as once inside a spine, AMPARs are trapped and do not readily escape. Therefore, exocytosis within spines is likely to be more efficient at delivering AMPARs to synapses than dendritic exocytosis (Kusters et al., 2013).

Following LTP-inducing stimuli, surface AMPAR levels increase quickly within seconds (Patterson et al., 2010) to minutes timescale (Kopec et al., 2006; Makino and Malinow, 2009) with a specific subset of AMPARs entering the synapse first. It is difficult to image directly AMPAR insertion into the plasma membrane with high spatial and temporal precision. Nevertheless, many groups have undertaken this herculean task in which cell culture models of LTP and LTD paradigms have proved invaluable. In one system, glass coated with neurexin was used to facilitate the formation of hemisynapses with neurons that express sep-tagged AMPAR subunits (sep-GluA1, sep-GluA2, sep-GluA3), and total internal reflection microscopy (TIRF) was performed to precisely image AMPAR delivery to the plasma membrane following induction of LTP by electrical field stimulation (Tanaka and Hirano, 2012). The initial response to stimulation was exocytosis of GluA1 homomers close to regions enriched with PSD-95, which was followed by a delayed exocytosis of GluA2 at peripheral sites. The slowest receptor to join was GluA3, again being inserted at peripheral sites, likely as GluA2/3 heteromers.

This result agrees with the significant body of data suggesting that the first receptors to be inserted at the synapse following LTP induction are calcium-permeable AMPARs (CP-AMPARs; Jonas and Burnashev, 1995). These are GluA2-lacking heteromeric receptors composed primarily of GluA1/3 subunits and are only transiently maintained at the synapse (Plant et al., 2006). Soon after LTP induction, synaptic recruitment of CP-AMPARs can add to the Ca^2+^ flow through synaptic NMDARs, triggering intracellular calcium-dependent signalling cascades that stabilize the newly-gained increases in synaptic strength (Park et al., 2018). Under basal conditions, C-tail interactions restrict GluA1-containing AMPARs from entering the synapse (Shi et al., 2001), but upon LTP stimuli they gain access to potentiate the synapse. GluA1-containing AMPARs thus act as the molecule underlying the expression of early LTP.

LTD is characterised by a drop in synaptic strength, and this is primarily mediated by the removal of AMPARs from synaptic sites involving dynamin-dependent endocytosis. Endocytic zones (EZ) are close to the PSD (Blanpied et al., 2002; Rácz et al., 2004), and disrupting these structures leads to a loss of synaptic AMPAR (Lu et al., 2009). However, the location of endocytosis associated with LTD may be variable. There is evidence from chemically induced LTD experiments in cultured neurons that synaptic AMPAR loss is preceded by endocytosis of the extrasynaptic pool of receptors (Ashby et al., 2004) and/or a suppression of exocytosis, leading to overall surface depletion of AMPARs (Fujii et al., 2018). Both studies induced LTD *via* NMDA application, and the differences between the two may reflect the differences in the AMPAR subunit observed, with Ashby et al. (2004) imaging sep-GluA2 and Fujii et al. (2018) imaging sep-GluA1. Loss of synaptic AMPAR is likely mediated by a disruption to the binding between AMPARs and their scaffolds, TARPs and PSD-95, allowing AMPARs to diffuse out of their synaptic domains (Bats et al., 2007). The interaction between AMPARs and TARPs is regulated by the phosphorylation state of TARPs (Tomita et al., 2007; Sumioka et al., 2010). Moreover, TARP phosphorylation has been shown also to alter the binding of the TARP stargazin (STG) to other adaptor proteins, and a mutant STG that does not bind AP-2 prevents NMDA-induced GluA2 endocytosis (Matsuda et al., 2013). For readers with a desire for more, please see a comprehensive review on AMPAR endocytosis by Hanley (2018).

### Heterosynaptic Changes in AMPARs

In general, it is assumed that LTP is synapse-specific, and as such, various stages of AMPAR recruitment and anchoring are thought to occur specifically at synapses that experienced activity change associated with LTP. However, as so often in biology, there are profound exceptions. LTP at one set of inputs has been known to cause plasticity at other, spatially distant inputs (Lynch et al., 1977), with the direction and amplitude of the heterosynaptic plasticity taking on different forms depending on the age, circuit, and preparation (reviewed in Chater and Goda, 2021). At the level of AMPAR subunits involved, synaptic stimulation was shown to drive synaptic insertion of GluA1, which was followed by heterosynaptic GluA1 insertion on a nearby dendrite surface (Patterson et al., 2010). These exocytosis events were restricted to within 3 μm of the stimulated spines and quickly returned to baseline levels when stimulation was discontinued. These receptors likely serve to replenish the extrasynaptic pool of receptors and/or provide the additional source AMPARs for the current round of plasticity. A modelling study also predicted that LTP and LTD have effects on nearby synapses (Antunes and Simoes-de-Souza, 2018). Simulating a thousand AMPARs diffusing on a small region of dendrite, homosynaptic LTP caused heterosynaptic depression, and the opposite was also true, with LTD at some spines leading to increased AMPARs at neighbouring synapses. The rules underlying heterosynaptic plasticity warrants further clarification along with mechanistic insights.

### AMPAR Distribution Across the Cell

**Figure 2:**
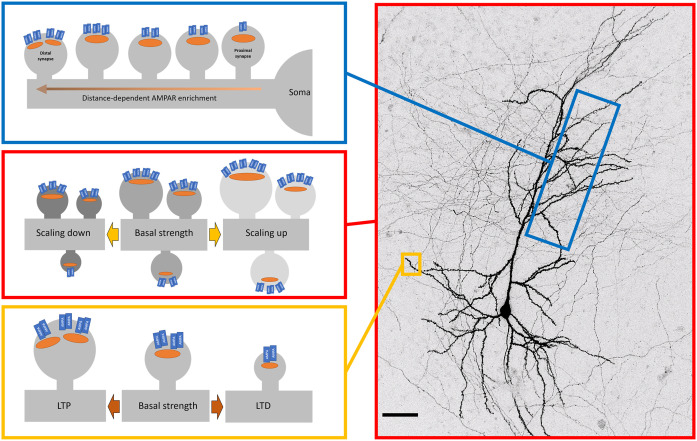
Patterns of AMPAR organisation and plasticity across the neuron. Surface AMPAR distribution is regulated by plasticity across different spatial levels. Top—in CA1 neurons, synaptic AMPAR levels increase along dendrites as the distance from the soma increases. This distance-dependent scaling offsets the dendritic filtering of distal inputs. Middle—Cell-wide AMPARs are modulated by changes in global activity levels. Blocking activity causes a compensatory increase in synaptic AMPAR levels (scaling up). Similarly, the increasing activity causes a down-scaling of AMPARs. These changes are multiplicative, thereby retaining the relative strengths encoded in synapses. Bottom—Activity-dependent trafficking of AMPARs. High-frequency stimulation increases levels of synaptic AMPARs that is one of the key cellular mechanisms of long-term potentiation (LTP). Low frequency simulation leads to loss of synaptic AMPARs (long term depression, LTD).

So far we have discussed the surface population of AMPARs at the level of individual synapses and their neighbouring extrasynaptic regions. However, beyond local dendritic branches, there is a structure of AMPAR distribution that manifests at larger scales along the dendritic distance (Andrásfalvy and Magee, 2001; see Figure 2). In hippocampal CA1 neurons, different subcompartments of the dendrite have different levels of AMPAR, with stratum radiatum (SR) having higher levels than stratum lacunosum-moleculare (SLM; Nicholson et al., 2006). The difference in synaptic AMPAR levels between SR and SLM in itself may be explained by the differences in the source of incoming axons and their relative roles in generating local dendritic spikes to boost information arriving in SLM (Wei et al., 2001; Jarsky et al., 2005). However, within SR, distal synapses have more AMPARs on average than proximal synapses (Nicholson et al., 2006; Shipman et al., 2013). A similar pattern was found for CA1 basal dendrites in stratum oriens (Menon et al., 2013). The synaptic AMPAR gradient has been measured by immunogold labelling (Nicholson et al., 2006), dendritic patching (Smith et al., 2003; Shipman et al., 2013), and glutamate uncaging (Smith et al., 2003) with largely consistent results. This distance-dependent scaling acts to counteract the filtering properties of dendrites (Magee and Cook, 2000) and facilitates the contribution of distal inputs to dendritic integration (Williams and Stuart, 2003).

What are the molecules required to create this pattern? Animals lacking the voltage-gated potassium channel Kv4.2 do not show this distance-dependent scaling in CA1 (Andrásfalvy et al., 2008), and deletion of the AMPAR accessory protein cornichon-2 (CNIH-2) selectively disrupts the distal inputs whilst leaving the proximal ones intact. Moreover, selective knockdown of GluA2 resulted in a reversal of distance-dependent scaling of the remaining AMPARs (Shipman et al., 2013). The distance-dependent scaling of AMPARs may be a feature unique to hippocampal CA1 pyramidal neurons. Although further studies may reveal similar scaling in other neuron types in other brain regions, in the neocortex at least, one study found no distance-dependence for synaptic AMPARs in pyramidal neurons (Williams and Stuart, 2002).

### Phosphorylation of AMPARs and the Tangled Tails

Phosphorylation of AMPARs plays a critical role in multiple types of plasticity (Carvalho et al., 2000; Wang et al., 2005; Purkey and Dell’Acqua, 2020). All four AMPAR subunits are substrates for phosphorylation by a host of protein kinases (Huganir and Nicoll, 2013). Whilst GluA1–4 are structurally similar, they differ most in the C-terminal domain (CTD) which is the primary interaction site with the PSD machinery and where several potential phosphorylation sites are also located. Consequently, much effort has been put into understanding the basis for CTD phosphorylation and its contribution to LTP, LTD, and homeostatic plasticity.

For GluA1, phosphorylation of serine 831 and 845 residues by PKA play key roles in LTP and its synaptic insertion (Esteban et al., 2003). Mice mutants with both loci changed to alanine show impaired LTP (Lee et al., 2003). The level of GluA1 S845 phosphorylation is linearly related to the surface fraction of GluA1 (Oh et al., 2006). Phosphorylation at this site responds bidirectionally to chem-LTP (≈60%) and chem-LTD (≈10%) from the resting state phosphorylation level of ≈15%. However, a more recent study found that less than one per cent of GluA1 is phosphorylated at either serine 831 or 835 under basal conditions (Hosokawa et al., 2015). At these levels, assuming 100 AMPARs/synapse, only one in six synapses contains a phosphorylated GluA1 at this residue. Another recent study however has found that 15%–20% of GluA1 is phosphorylated, and this increases to ≈60% following chem-LTP (Diering et al., 2016), which is in line with previous work. What can explain this discrepancy? For one, the studies induced chem-LTP with different stimuli. Another factor is the method to measure phosphorylation. Hosokawa et al. (2015) used a novel SDS-PAGE technique, while Oh et al. (2006) and Diering et al. (2016) used immunofluorescence and/or surface biotinylation.

With respect to LTD, dephosphorylation of GluA1 results in its removal from the synapse (Lee et al., 1998; Beattie et al., 2000). Again, S845 is implicated, as mice carrying a point mutation where serine is replaced by alanine (S845A) have reduced LTD (Lee et al., 2010). Similarly, double phosphomutants (replacing both S831 and S845 with alanine) have perturbed LTD, whilst S831A mutation alone does not affect LTD (Lee et al., 2003).

AMPAR phosphorylation is also important for homeostatic forms of plasticity (see below). PKA phosphorylation of GluA1 S845 has been implicated in the scaling up of synaptic AMPARs in cultures (Diering et al., 2014; Kim and Ziff, 2014) and *in vivo* (Goel et al., 2011). The coupling between GluA1 and PKA is mediated by the scaffold protein AKAP5, and upon scaling down PKA diffuses away from the synapse (Diering et al., 2014).

Recent work has fuelled the debate on the function of the CTDs of GluA1 and GluA2, despite the seemingly crucial role for synaptic plasticity of GluA1 S845 that sits on the CTD. A knock-in mouse where endogenous GluA1 was replaced with GluA1 containing the CTD of GluA2 (GluA1A2CTD) showed no changes in basal synaptic transmission but LTP was entirely abolished (Zhou et al., 2018). This LTP deficit could be rescued by overexpression of a GluA2 subunit containing the GluA1 tail. A follow-up study from the same group found that in older mice there was still some LTP intact, and the involvement of GluA1 CTD was dependent on age and how LTP was induced (Liu et al., 2020). On the other hand, a single-cell approach, utilizing a cre-recombinase dependent knockdown of GluA1–3, and subsequent overexpression of GluA1A2CTD, found no changes in LTP (Díaz-Alonso et al., 2020). Earlier work from the same group similarly found no requirement for the GluA1 subunit for LTP (Granger et al., 2013). How LTP is induced seems to be a critical factor in the apparent requirement for GluA1.

One possible explanation for these discrepancies could be the experimental method used for LTP induction, with field stimulation vs. pairing protocol. Paired recording, in which the activity of the postsynaptic neuron can be deliberately controlled, for example by current injection to elicit spikes at different times relative to the presynaptic stimulation, may engage a subtly different set of synaptic plasticity mechanisms than field stimulation in which the postsynaptic neuron is left to naturally respond to the simulation.

### Life Without AMPARs—Genetic Manipulations

A popular means of testing the role of a particular protein is to remove it entirely from the system and see what happens. Such approaches have provided a wealth of information and critical insights into AMPAR functions and mechanisms, but not without contradictions. Here we briefly summarize key knock-out studies of AMPAR subunits.

GluA1-lacking mice (*Gria1*^−/−^) show disruption to LTP, including a deficit in associative LTP (Zamanillo et al., 1999; Hoffman et al., 2002) and loss of pairing-induced LTP (Jensen et al., 2003; Shimshek et al., 2017). However, in younger mice (Jensen et al., 2003) and with a modified LTP-induction protocol (Hoffman et al., 2002), it is still possible to induce LTP in the absence of GluA1. The spatial working memory (SWM) deficit displayed by GluA1 KO mice can be partially rescued by re-expression of GluA1 (Shimshek et al., 2017). Fascinatingly, on the GluA1 KO background, overexpression of GluA1 subunits lacking their CTDs or additional removal of GluA2 could partially restore LTP but not the deficit to SWM (Shimshek et al., 2017). These observations not only underscore the disjoint between the mechanisms of LTP studied *in vitro* and the behavioural assessment of SWM but reveal a striking interplay of GluA1 and GluA2 subunits and support the importance of the GluA1 CTD for synaptic plasticity.

Another fruitful approach to studying AMPAR function has been to knock down AMPAR subunits in isolated single cells and examine the effects on baseline synaptic function and synaptic plasticity. The Nicoll lab capitalised on mice floxed for GluA1–3 to knock-down all three subunits by expressing Cre-recombinase in CA1 neurons. These cells showed no AMPAR EPSCs, a massive reduction in glutamate-induced currents, with no effect on NMDARs or dendritic branching and spine number (Lu et al., 2009). Removal of GluA3 alone caused a modest reduction in AMPAR currents, whilst removal of GluA1 massively depleted surface and synaptic AMPARs. The authors concluded that AMPARs in CA1 neurons are therefore almost entirely GluA1/2 heteromers. Upon further examining the dependence of LTP on specific subunits, however, to many’s surprise, cells with GluA1 alone, GluA2 alone, or even solely an artificial kainate receptor were all capable of expressing normal LTP (Granger et al., 2013). The one requirement was an extrasynaptic pool of receptors. This finding adds to the evidence that much of the enhancements in synaptic strength during LTP come from lateral diffusion and synaptic recruitment of AMPARs from an extrasynaptic pool. An analogous approach to LTD also revealed a similar lack of subunit specificity for the removal of synaptic AMPARs (Granger and Nicoll, 2014). Collectively, these results suggest the flexibility of the system underlying synaptic performance, at least in defined *in vitro* plasticity paradigms.

Early experiments on GluA2 KO animals found an enhancement of LTP (Jia et al., 1996), presumably generated by increased calcium entry into postsynaptic compartments by CP-AMPARs. GluA2 deficits have been linked to disease, including autism spectrum disorders and intellectual disability (Salpietro et al., 2019). In contrast, a recent study, working on CA1 neurons from a fragile X mouse model, found a transient decrease in GluA2-lacking AMPARs compared to control neurons, early in development (Banke and Barria, 2020). This may lead to reduced calcium fluxes into these cells during this early phase to potentially hamper synapse formation and dendritic outgrowth. Therefore, a balanced expression of GluA1 and GluA2 might be desired for proper brain development and function.

### Global Changes in AMPAR Across the Dendritic Arbor

Neuronal activity patterns cover a broad swathe, from high-frequency bursts to periods of quiescence with no spiking at all. Within such a dynamic activity regime, neurons are capable of maintaining their own firing rate within a range by engaging a number of homeostatic mechanisms that include altering synaptic AMPAR complement (see Figure 2). Now classic studies using dissociated cultures of visual cortical or spinal neurons have demonstrated that silencing of network activity by the Na^+^channel blocker TTX, for 48 h produced an increase in their synaptic AMPAR levels as measured by miniature excitatory postsynaptic currents (mEPSCs) and immunocytochemistry (O’Brien et al., 1998; Turrigiano et al., 1998). This “synaptic scaling” was cell-wide and multiplicative, where the relative differences in synaptic strengths were maintained, thereby providing a plausible mechanism for preserving the information content of the network. Further experiments found that this regulation was bidirectional, as treatment with a GABA_A_ antagonist (bicuculline) to elevate network activity caused a concomitant decrease in synaptic strengths globally. This result drove a great deal of interest, and the core result has been replicated in various cortical and hippocampal dissociated cultures (e.g., Cingolani et al., 2008), organotypic hippocampal slices (e.g., Kim and Tsien, 2008), acute slices (e.g., Huupponen et al., 2007), and *in vivo* (e.g., Desai et al., 2002). The homeostatic scaling of AMPARs is regulated at least in part cell autonomously, as the scaling can be observed upon inhibiting activity in single cells (Ibata et al., 2008).

Many studies have since explored the temporal pattern of synaptic changes in response to activity deprivation. GluA2 tagged with YFP (whose fluorescence depends on the local pH, similarly to sep) demonstrated that AMPAR upregulation occurs as quickly as within 1 h following TTX application (Ibata et al., 2008). In a set of beautiful experiments, the authors locally perfused TTX onto the soma of individual neurons for 4 h, showing that this was sufficient to increase synaptic AMPAR accumulation. Further experiments linked somatic calcium entry to the changes in synaptic AMPAR *via* a reduction in calcium/calmodulin-dependent protein kinase type IV (CaMKIV) signalling (Ibata et al., 2008).

Bissen et al. (2021) used entorhinal-hippocampal organotypic slices and expansion microscopy to examine the precise requirement for AMPAR trafficking after de-innervation from the entorhinal input. Like previous work (Tan et al., 2015) they identified glutamate receptor interacting protein 1 (GRIP1) as a key regulator of AMPAR trafficking following activity deprivation. GRIP1 is also required for LTP-mediated AMPAR insertion (Tan et al., 2020) for GluA1–3, and GRIP1 KO animals show a deficit in learning and memory, but unaffected LTD (Tan et al., 2020). These findings highlight a recurrent theme across different forms of synaptic plasticity that some of the molecular mechanisms are shared despite the differences in the context in which AMPAR levels are altered.

Much of the pioneering insights on homeostatic regulation of AMPARs have come from work in cultured neurons but *in vivo* studies have continued to extend our knowledge of how different activity levels impact synaptic strengths. Loss of an input due to injury or circuit dysfunction in a diseased state can lead to brain consequences similar to those of activity deprivation as studied in culture. The visual cortex has proved an excellent area to investigate homeostatic regulation due to the relative ease of silencing visual input by eye closure (Desai et al., 2002), intraocular injection of TTX (Gainey et al., 2009), or retina lesions (Keck et al., 2013). Desai et al. (2002) found that functional synapse development as monitored by mEPSC amplitude and frequency was altered by either raising the animals in darkness or 2 days of monocular deprivation, the latter of which scaled up mEPSC amplitude. Another study demonstrated that this ability to undergo *in vivo* scaling up was confirmed also in the adult cortex following the same 2 days of light deprivation (Goel and Lee, 2007) and fully reversible by one day of light. This study found a correlation between the levels of phosphorylated GluA1 S845, synaptic CP-AMPARs, and mEPSC amplitude increases, linking dissociated culture observations to synaptic scaling *in vivo*. Synaptic scaling has also been demonstrated in the auditory cortex, where hearing loss initially causes a drop in activity in the primary auditory cortex (A1), which then responds by scaling up mEPSC amplitudes in layer 2/3 pyramidal neurons over 3 days (Teichert et al., 2017). Notably, consistent with the requirement for a cytokine TNFα that is released from glial cells in scaling up of mEPSCs in dissociated cultures, TNFα KO mice showed impaired scaling up of mEPSCs upon activity deprivation (Teichert et al., 2017). Therefore, TNFα signalling pathway is important in controlling synaptic AMPAR content both *in vitro* and *in vivo* (see Heir and Stellwagen, 2020, for a comprehensive review of TNFα’s role in homeostatic plasticity).

As a side note, a large body of mechanistic dissection of *in vivo* synaptic homeostasis has focussed on NMDARs, and GluN2B subunit, in particular, seems to be the heavily implicated subtype (e.g., Chung et al., 2017; Rodriguez et al., 2019). The readers are referred to Lee and Kirkwood (2019) for an excellent and comprehensive review of *in vivo* homeostatic plasticity.

### AMPAR Dynamics *In vivo*

Many measurements of neuronal activity depend on synaptic AMPARs, and recently there has been a push to directly monitor fluorescently labelled AMPAR behaviour *in vivo*. For this, sep-tagged receptor subunits have been invaluable.

Using sep-GluA1, a recent study has estimated the mobile fraction of AMPARs *in vivo*. The authors find that 50% of sep-GluA1 recovered after bleaching, revealing a relatively high proportion of fluid AMPARs (Chen et al., 2021). Consistently, whisker stimulation drives NMDAR-dependent increases in the surface levels of sep-GluA1 in neurons in the barrel cortex, both in synapses (Makino and Malinow, 2011) and nearby dendritic shafts (Zhang et al., 2015). In animals with trimmed whiskers, sep-GluA2 is enriched in spines, matching the *in vitro* homeostatic role that has been identified for this subunit (Gainey et al., 2009). These are all overexpression experiments, with varying tweaks to the combination of AMPAR subunit expressed (e.g., expressing sep-GluA1 alongside myc-tagged sep-GluA2 to facilitate the formation of heteromeric receptors over biasing the generation of homomers: Chen et al., 2021). Recently, advances in genome editing technologies have made the imaging of endogenous receptors more viable (e.g., vSLENDR: Nishiyama et al., 2017). Nevertheless, even tagging endogenous AMPARs can lead to alterations in expression, a situation that is not surprising given the substantial size of the popular fluorescent proteins. For example, a KI mouse expressing sep-GluA1 shows significantly decreased synaptic GluA1, with possible compensatory increases in GluA2 and GluA3 (Graves et al., 2021). On the other hand, these mice show normal LTP, bidirectional synaptic scaling, and no difference in the behaviour compared to WT mice across a variety of behavioural paradigms. Moreover, the authors were able to image hundreds of thousands of sep-GluA1 labelled synapses *in vivo*, and following a whisker stimulation protocol, detect increases in sep-GluA1 signal in the barrel cortex. Combining these direct monitoring of AMPARs at individual synapses with fast activity readouts (for example red calcium/voltage indicators) hints at datasets that will truly enhance our understanding of brain function from synapse to spike, to circuits and behaviour.

## Conclusion

This review has only scratched the surface of our knowledge about AMPARs, and wherever possible, we have directed the readers to reviews that further explore the subtopics in detail. Regardless, still, significant questions remain to be answered.

The precise roles of specific AMPAR subtypes in learning and memory, if any, are still unclear. Much of the data and the conflicts therein have come from acute slice experiments employing a variety of plasticity induction protocols. Genetic manipulation using KO mice or inducible KD, where they exist, often remove a particular AMPAR subunit or its regulatory protein across the brain or in a subset of neurons in a fairly uniform manner, whereas individual proteins may play subtly different roles in say the hippocampus and the cortex and its loss may result in complex compensation that could mask the real function. Studies that have removed all the AMPARs from neurons to replace them one by one are wonderfully elegant but arguably artificial. Overexpression of receptors, and especially of tagged receptors, may also lead to artefacts. These may go some way to explain current discrepancies in the field. Overall, despite the large degree of confounds, the field has made tremendous progress over the past several decades since the identification of AMPARs.

Having said that, why not go further? It would be very powerful and interesting to have optical control over different AMPAR subunits so that they could be reversibly deactivated/silenced on short time scales within the same cell. Steps towards this have been demonstrated by manipulating glutamate receptors using photoswitchable tethered ligands (Volgraf et al., 2006; Kienzler et al., 2013) that are sensitive to different wavelengths of light. This hypothetical arrangement would allow a better dissection of the requirements for particular subunit combinations during different stages of plasticity/memory formation. Given the challenges of synaptic measurements and manipulations *in vivo*, exploiting simpler model systems may still yet be highly productive. As we collect more and more high-resolution data of synaptic structure and variation, AI-assisted data mining may be a useful tool to add to the AMPAR enthusiasts’ toolbox.

## Author Contributions

All authors listed have made a substantial, direct, and intellectual contribution to the work and approved it for publication.

## Funding

Research in authors’ laboratory is supported by the RIKEN Center for Brain Science, the Japan Society for the Promotion of Science Core-to-Core Program (JPJSCCA20170008).

## Conflict of Interest

The authors declare that the research was conducted in the absence of any commercial or financial relationships that could be construed as a potential conflict of interest.

## Publisher’s Note

All claims expressed in this article are solely those of the authors and do not necessarily represent those of their affiliated organizations, or those of the publisher, the editors and the reviewers. Any product that may be evaluated in this article, or claim that may be made by its manufacturer, is not guaranteed or endorsed by the publisher.
